# Structural Study of the Thermoelectric Work Units Encapsulated with Cement Paste for Building Energy Harvesting

**DOI:** 10.3390/ma17040926

**Published:** 2024-02-17

**Authors:** Ziqiang Lai, Yali Hao, Yongqi Wei, Anming She, Wu Yao

**Affiliations:** Key Laboratory of Advanced Civil Engineering Materials of Ministry of Education, School of Materials Science and Engineering, Tongji University, Shanghai 201804, China

**Keywords:** thermoelectric, cement paste, heat transfer, natural heat dissipation, building energy harvesting

## Abstract

Cement-based material encapsulation is a method of encapsulating electronic devices in highly thermally conductive cement-based materials to improve the heat dissipation performance of electronic components. In the field of construction, a thermoelectric generator (TEG) encapsulated with cement-based materials used in the building envelope has significant potential for waste heat energy recovery. The purpose of this study was to investigate the effect of cement-based materials integrated with aluminum heatsinks on the heat dissipation of the TEG composite structure. In this work, three types of thermoelectric work units encapsulated with cement paste were proposed. Moreover, we explored the effect of encapsulated structure, heat dissipation area, the height of thermoelectric single leg, and heat input temperature on maintaining the temperature difference between the two sides of the thermoelectric single leg with COMSOL Multiphysics. The numerical simulation results showed that under the conditions of a heat source temperature of 313.15 K and ambient temperature of 298.15 K, the temperature difference between the two sides of the internal thermoelectric single leg of Type-III can maintain a stable temperature difference of 7.77 K, which is 32.14% higher than that of Type-I and Type-II (5.88 K), and increased by 26.82% in the actual experiment. This work provides a reference for the selection and application of TEG composite structures of cement-based materials combined with aluminum heatsinks.

## 1. Introduction

Fossil fuels still dominate with oil, coal, and natural gas accounting for 82% of global energy supply [1]. In the face of the continuous growth of energy demand and the massive consumption of fossil energy, it is of great importance to solve the problem of the energy crisis when considering the sustainable survival and development of mankind. A thermoelectric generator (TEG) absorbs ambient thermal energy and converts it into electricity through the Seebeck effect [2] and has the characteristics of small size, high reliability, no pollutants, and feasibility in a wide temperature range [3]. Applying TEGs on building surfaces such as roofs [4], walls [5], windows [6], and roads [7] enables large-scale low-temperature waste heat energy harvesting.

In the scenario of continuous heating and the absence of effective thermal management of TEG systems, the overall temperature of the device escalates, leading to a notable decline in output performance. How to solve the problem of TEG thermal management has become a key issue in the field of building energy harvesting. Currently, the cooling methods of TEG systems mainly include forced water cooling, air cooling, and thermal management of phase change materials (PCMs). For example, Musleh et al. [8] modeled and simulated the electrical output performance of an external water-cooling TEG with a temperature difference of 10 °C between the two sides. And the results showed that the output power of a matched load of a TEG was about 18 mW. Gu et al. [9] developed an implantable TEG module designed for large-scale flue gas waste heat recovery and investigated the effect of cooling water temperature on the electrical performance of the TEG module. The results demonstrated that, under specific conditions where the flue gas temperature reached 139 °C, the flue gas flow rate was maintained at 3.4 m/s, the cooling water temperature was kept at 20 °C, and the 240 TEG modules exhibited optimal performance. It has an open circuit voltage of 856.3 V and an output power of 150.58 W. However, the water-cooling method requires additional energy consumption based on the installation load of complex pipes. Murčinková et al. [10] tested the output performance of the stove-power TEG under forced air cooling with a temperature difference of 94 °C and an output power of 1.5 W. Chen et al. [11] also simulated forced convection cooling of natural wind speed by Taguchi optimization and operated it at low mass waste heat temperature to further improve TEG performance. Nevertheless, the installation of fans in practical applications often encounters challenges such as noise, structural design, and maintenance issues, leading to limitations in their applicability. The use of PCMs is more ideal for thermal management of building TEG systems. For instance, Madruga et al. [12] proposed an enhanced micro energy collector for PCM/TEG and pointed out that the porosity of metal foam accelerates heat transfer and allows the volume of a higher-temperature heat storage device to effectively collect more energy from the surrounding environment. Rezania et al. [13] then combined PCMs and copper foam to reduce the temperature of the cold side of a TEG and prevent the hot side of the TEG from overheating. However, the thermal conductivity of PCM is low, and its low density makes the volume of PCM change greatly when it absorbs or releases heat, which may affect the stability of the TEG. In addition, the large-scale use of PCMs for TEG refrigeration is obviously not economical.

We noted that cement-based materials can effectively reduce the operating temperature of electronic devices and improve the stability of electronic devices in service. And cement encapsulation has the advantages of low cost, good plasticity, light weight, and so on. By changing the composition and structure of the cement to form a composite material, researchers have further improved its thermal conductivity. For example, adding thermal conductive fillers [14], nanomaterials [15], or changing the microstructure of the cement matrix can significantly change the thermal conductivity. Cement-based materials and thermoelectric materials have a good match in material compatibility, good thermal conductivity, easy availability, and wide applicability. Furthermore, cement-based materials are needed in most infrastructure. But most research has focused on the use of cement-based materials as tools to transfer heat to TEGs or as materials for thermoelectric conversion. For example, Mona et al. [16] studied the potential of pavement energy harvesting by comparing the use of TEG modules combined with heat transfer of asphalt and cement blocks. Wei et al. [17] proposed to use the dry-pressing method to prepare and assemble large thermoelectric modules of cement-based composite materials for road surface energy collection. The results show that cement-based composite materials can also reduce the temperature by 1–3 °C, which can significantly reduce the surface temperature and alleviate the urban heat island effect. However, even the large-scale use of thermoelectric cement-based materials still cannot get rid of the reality of ultra-low energy conversion efficiency. Consequently, it is particularly essential to seek new cement-based material utilization schemes for traditional TEG modules. Félix-Herrán et al. [18] conducted a characterization system study for heat energy to electricity conversion from concrete by TEG modules. Similarly, Win et al. [19] proposed that applying TEGs to a typical structural facade using 25 cm thick reinforced concrete, and a peak power generation of 100.0 mW/m^2^ was obtained. This also validated the feasibility of cement-based materials for TEG heat dissipation. Nevertheless, those studies ignored that different structural combination forms will also affect the electrical and thermal properties of TEGs.

Considering the literature review and to the best of the authors’ knowledge, there are few studies systematically exploring the relationship between thermoelectric harvesters and cement-based materials. Herein, the forms of the TEG composite structure of a cement-based material integrated with an aluminum heatsink will be systematically discussed. And the dynamic characteristics and temperature difference maintenance effect of cement-based TEG power generation systems will be investigated. This paper aims to explore the thermal regulation capabilities of thermoelectric work units encapsulated with cement paste, evaluating factors including encapsulated structure, thermoelectric leg height, heat dissipation area, and heat input temperature. Taking temperature difference as the metric, we will analyze how different parameters impact maintaining the temperature difference of thermoelectric work units. For this purpose, we simplified the TEG device structures and proposed three thermoelectric work units—Type-I, Type-II, and Type-III—encapsulated with cement paste. Computational studies were conducted with COMSOL Multiphysics to assess the thermal regulation performance of these work units across various encapsulated structures, thermoelectric leg heights, heat dissipation areas, and heat input temperatures. Considering the internal structure of the work units and the effect of free water on cement matrix heat capacity, validated experiments were conducted to evaluate their practical application in thermal management for thermoelectric work units. Consequently, to intuitively evaluate the structural effect of TEG thermal performance, cold-pressed thermoelectric single legs were used to fabricate corresponding work units for recording real-time thermal electromotive force which can be calculated as temperature difference. The actual power generation capacity of thermoelectric work units in series was analyzed.

## 2. Design of Cement-Based Thermoelectric Work Units

**Figure 1:**
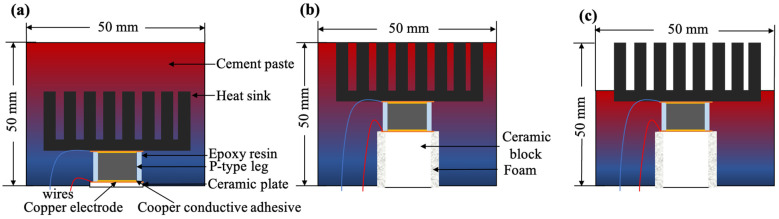
Thermoelectric work units: (**a**) Type-I; (**b**) Type-II; (**c**) Type-III.

**Figure 2:**
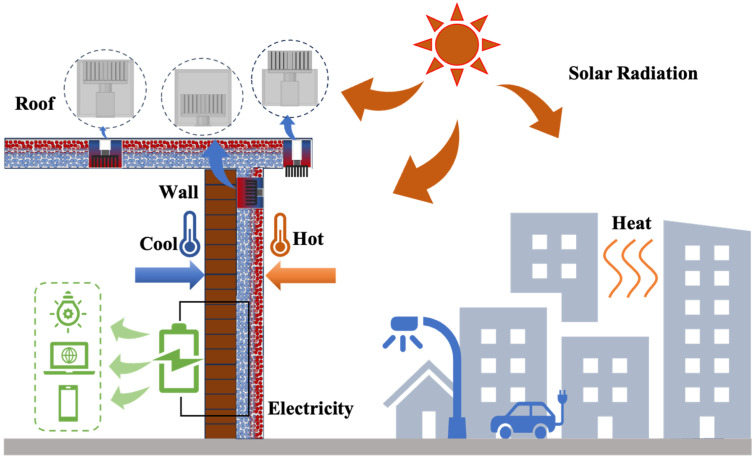
Schematic diagram of the actual use scenario of the thermoelectric work units.

The schematic of work units including Type-I, Type-II, and Type-III is shown in Figure 1a, Figure 1b, and Figure 1c, respectively. For the reference prototype of Type-I [19], the TEG has its hot side directly exposed to the heat source, without a connected heat transfer block/pipe, and the cold side of the TEG directly contacts the cement-based material. It is directly used for surface structures such as vertical walls or roofs. The simplified model of Type-II is derived from the geothermal energy collection system [20]. The TEG module is deeply buried underground, and heat transfer tool—heat transfer block/pipe—is required for heat transfer. The heat passes through the TEG and generates electricity, which is then dissipated from the cold side underground. Type-II is used for the interior of the wall or roof. The thickness of the heat transfer block/pipe depends on the actual situation. The heat passes through the heat transfer block/pipe and then contacts the hot side of the TEG, and the cold side contacts the cement-based material. Type-III is a composite heat dissipation model that integrates the heat dissipation advantages of cement-based materials and metal materials. A heat transfer block is utilized to transfer heat to the hot side of the TEG. This model transfers heat to cement-based materials through the substrate of the aluminum heatsink and dissipates heat at the same time with the help of the refined fins of the heatsink. It is suited for eaves, roofs, and other structures, and the Type-III model is arranged throughout the entire structure. The actual application usage scenario of the three thermoelectric work units can be seen in Figure 2.

Each model is divided into two parts, encapsulated structure and encapsulating cement paste. The inner structure contains the heat transfer block, thermoelectric single leg, and aluminum heatsink. The thermal conductor is a ceramic plate for Type-I, while it is a ceramic block for Type-II and Type-III. The insulation foam is used to reduce heat loss in the heat transfer process. Pure cement paste is employed to encapsulate the inner TEG structures.

The thermoelectric single leg is a cylinder 10 mm in diameter and 8 mm in height. The ceramic plate (15 mm × 20 mm × 1 mm) and ceramic block (15 mm × 20 mm × 23 mm) are used as thermal conductors. The insulation foam (thickness = 1 mm) was wrapped around the ceramic block to enhance thermal insulation. The dimensions of the finned aluminum heatsink are presented in Table 1. A cubic silicone mold (50 mm × 50 mm × 50 mm) was used for cement paste casting.

## 3. Numerical Method

### 3.1. Control Equations

Heat conduction, radiation, and air natural convection are involved in this section. The COMSOL Multiphysics 6.0 can be used to simulate the working process of work units in a transient state. The test models used for the computational study are simplified and consist of a P-type bismuth telluride (Bi_2_Te_3_) leg, ceramic plate (or block), insulated foam, heatsink, and cement paste. Due to the simplified processing of the model, the copper electrode, copper conductive adhesive, and wires are neglected in the models. The properties of corresponding materials come from the material library of COMSOL Multiphysics. The working process of the work units can be developed by the Fourier law of heat conduction, Seebeck effect, Peltier effect, Joule heat, and thermodynamic energy. The governing equations can be expressed as follows [21,22]:

(1) $$\rho C_{P}\frac{\partial T}{\partial t}+\nabla \vec{q}=Q$$

(2) $$\vec{J}=\sigma (-\nabla \varphi +N\alpha \nabla T)$$

(3) $$\vec{E}=-\nabla \varphi +N\alpha \nabla T$$

(4) $$\vec{q}=N\alpha T\vec{J}-\kappa \nabla T$$

where ρ is the density (kg/m^3^), $C_{P}$ is specific heat capacity (kJ/(kg·K)), *T* is temperature (K), $\vec{q}$ is heat flux vector (W/m^2^), *Q* is heat (W/m^2^), $\vec{J}$ is electric current density vector (A/m^2^), σ is electrical conductivity (S/m), *N* is the number of Bi_2_Te_3_ legs, $\vec{E}$ is electric field density vector (V/m^2^), φ is electric potential (V), α is Seebeck coefficient (V/K), κ is thermal conductivity (W/(m·K)). Substituting Equations (2)–(4) into Equation (1), the transient governing equation of the thermoelectric single leg can be expressed as [21]:

(5) $$\rho C_{P}\frac{\partial T}{\partial t}+\nabla [-\kappa \nabla T-N\alpha T\sigma (-\nabla \varphi +N\alpha \nabla T)]=\sigma {\left(-\nabla \varphi +N\alpha \nabla T\right)}^{2}$$

The surface radiation to the environment is taken into account. The governing equation can be written as [23]:

(6) $$\vec{q_{r}}=\varepsilon \delta (T^{4}-T_{amb}^{4})$$

where $\vec{q_{r}}$ is radiant heat vector, ε is surface emissivity (ε = 0.9), δ is Stefan–Boltzmann constant (W/(m^2^$\cdot K^{4}$)), and *T_amb_* is the ambient temperature. The temperature of the work unit is different from that of the surrounding air, and there is natural convection on the surface of the work units. The air’s natural convection can be determined as follows [23]:

(7) $$\vec{q_{c}}=\alpha_{c}(T-T_{amb})$$

where $\vec{q_{c}}$ is convective heat flux vector, $\alpha_{c}$ is the convective heat transfer coefficient (W/(m^2^·K)), and $\alpha_{c}$ is 5 W/(m^2^·K). The total boundary heat flux is the sum of the radiant heat flux and the convective heat flux.

### 3.2. Boundary Condition

The numerical models are performed with the dynamic characteristic of a thermoelectric coupled physical field. The setting of boundary conditions refers to the actual test conditions of the thermoelectric work units. To comprehensively analyze the dynamic change temperature difference curves of the three thermoelectric work units, the calculation time is fixed at a steady state—8000 s—where the temperature difference remains relatively stable. It is assumed that the heated area is only the ceramic contact surface to ensure that the heat source of the overall temperature increase in the thermoelectric work units is through the thermoelectric leg. Although, under actual conditions, the heat source will not be present at only a single designated contact surface. The system’s initial temperature is uniformly set based on typical indoor ambient conditions. To assess how the height of the thermoelectric single leg impacts temperature difference maintenance, various units with different thermoelectric leg heights were established. Similarly, to explore the influence of heat dissipation area, thermoelectric work units equipped with heatsinks of varying sizes are considered. Notably, solar radiant heat flux is excluded as the system’s primary heat source due to the complexity of factors—such as material refractive indices and ambient temperature—that influence the calculated temperature at the irradiated hot side. Instead, this study focuses on the direct heat input temperature applied to the ceramic contact surface. Different temperatures and hot side configurations were tested to elucidate the effects of input temperature on temperature difference maintenance. Under natural conditions, heat transfer mechanisms like heat conduction, air natural convection, and radiation occur simultaneously, resulting in heat exchange at all system boundaries. It is worth noting that in the practical scenario, the thermoelectric unit is surrounded by concrete rather than air. However, the simplified model of the thermoelectric work units still demonstrates its dynamic temperature retention effect when heated. Given the relatively consistent packaging conditions of the thermoelectric legs across the three units, the model is appropriately streamlined, excluding considerations of contact thermal resistance and contact resistance. The total boundary conditions of the numerical simulation are presented as follows:

(1) The dynamic simulation is assumed and the calculation time of heat transfer is programmed to 8000 s.
(2) The heat source contacting face is located at the bottom of the work units (the ceramic heated area is 15 mm × 20 mm).
(3) The initial and ambient temperature of the systems: t = 0, T = T_0_ = T_amb_ = 298.15 K.
(4) The model is determined as Type-III, while the height of the thermoelectric single leg is 2 mm, 4 mm, 6 mm, and 8 mm, respectively.
(5) The model is determined as Type-III, and the heatsink is S1 (22 mm × 28 mm × 1 mm), S2 (40 mm × 40 mm × 1 mm), and S3 (finned aluminum heatsink), respectively.
(6) The heat input temperature is 303.15 K, 308.15 K, and 313.15 K at the bottom of Type-III.
(7) No surfaces are thermally insulated.
(8) The thermal and electrical contact resistances are not taken into account.

### 3.3. Model Validation

**Figure 3:**
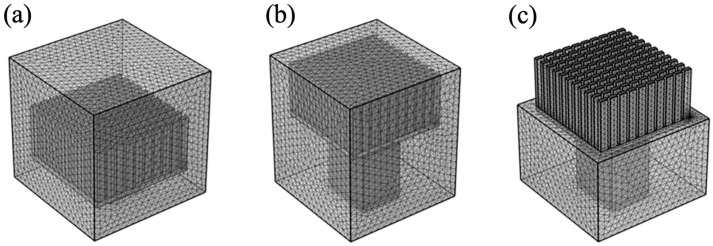
Schematic diagram of grid division: (**a**) Type-I; (**b**) Type-II; (**c**) Type-III.

**Figure 4:**
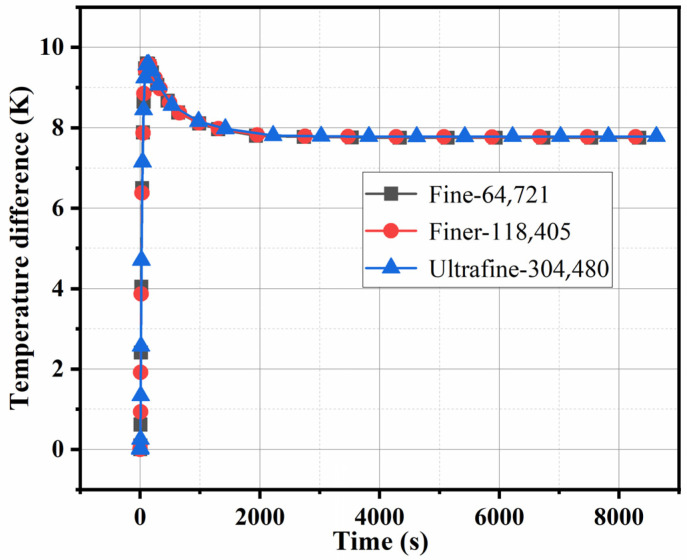
Grid analysis for various mesh numbers.

According to the physical structure of the system, COMSOL Multiphysics is used to establish the corresponding physical models. The numerical simulation was performed in the transient state under the condition that the ambient and heat input temperatures were 298.15 K and 313.15 K, respectively. When the work unit has a stable thermal electromotive force indicating its good thermal management capability, the temperature difference of the thermoelectric single leg can be calculated according to the Seebeck effect. Hence, the temperature difference of the work units with different parameters was investigated to illustrate the thermal management performances. The models are divided into tetrahedral structured grids. The schematic diagrams of grid division of Type-I, Type-II, and Type-III are presented in Figure 3a, Figure 3b, and Figure 3c, respectively. In the grid independence study, we calculated the temperature difference process under three different mesh numbers to ensure the accuracy of the numerical simulation. It can be observed from Figure 4 that no significant change occurred in the calculated temperature difference when the mesh numbers are changed. Increasing the density of the grid can obtain more accurate numerical solutions, but it requires more computer resources. When the number of refined meshes reaches a certain level, increasing the mesh density will only cause a small or no change in the calculation result. The numerical solution obtained at this time is the most accurate one available, that is, the grid-independent solution. The refiner grid is used for simulation calculation. The stable temperature difference is lower than 0.1%, which means the used meshing grid is reliable for a precise calculation. It is found that the temperature difference error of this work is lower than 0.1%, similar to published literature [24]. The following factors are taken into account for the heat transfer of thermoelectric work units that are encapsulated with cement paste: heat dissipation area, thermoelectric leg height, and the heat input temperature of the system.

## 4. Experimental

### 4.1. Materials and Properties

In this work, 42.5 ordinary Portland cement with an apparent density of 3.1 g/cm^3^ was used as a cementitious material. The commercial P-type Bi_2_Te_3_ powder was purchased from Qijin New Material Co., Ltd., Quanzhou, China, and the corresponding thermoelectric properties are shown in Table 2. Ceramic materials, copper conductive adhesive, epoxy resin, copper electrodes, wires, foam insulation, aluminum heatsink, and thermal silicone grease used in this study were purchased, and the detailed performance parameters of the involved materials are shown in Table 3.

### 4.2. Fabrication

**Figure 5:**
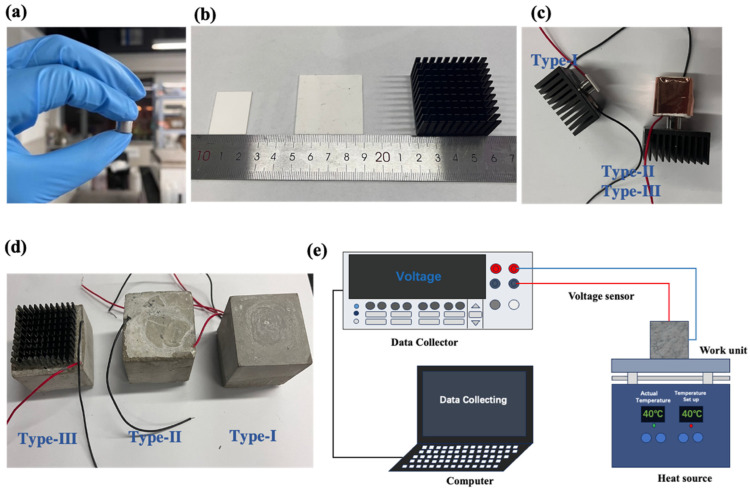
(**a**) Cold-pressed P-type bulk semiconductor; (**b**) heatsinks: S1 (22 mm × 28 × 1 mm), S2 (40 mm × 40 mm × 1 mm), and S3 (finned aluminum heatsink); (**c**) encapsulated structures of Type-I, Type-II, and Type-III; (**d**) Sample of Type-I, Type-II, and Type-III encapsulated with cement paste; (**e**) Schematic of the experiment set-up.

The raw thermoelectric Bi_2_Te_3_ powder was cold-pressed into a cylindrical thermoelectric single leg, as shown in Figure 5a. The different heatsinks and encapsulated structures are presented in Figure 5b,c. The schematic of the work units encapsulated with cement paste is shown in Figure 5d. The main structure of work units includes a thermoelectric single leg, thermal transfer plate (block), and finned aluminum heatsink. To change the position of the Bi_2_Te_3_ single leg in the cement matrix, ceramic bulk (15 mm × 20 mm × 23 mm) was used as a thermal conductor. It improves the position of the thermoelectric leg and increases the cement matrix space for heat transfer. The finned aluminum heatsink has an excellent thermal conductivity of 238 W/(m⋅K), which effectively reduces diffusion resistance and hastens thermal transfer. The ceramic block is wrapped with foam insulation to reduce heat loss. The ratio of water to cement (W/C) was kept constant at 0.3 in the fabrication of cement paste. Electrodes and thermoelectric legs were coated with epoxy resin to protect them from electrical failure during the 28 d cement hydration.

### 4.3. Experimental Procedure

To collect the thermal electromotive force data of Type-I, Type-II, and Type-III, a complete prototype of the thermoelectric power generation test system was established as indicated in Figure 5e. The experimental setup included a heating platform, a multifunctional data collector, and a computer. The heating platform was preheated to 313.15 K. The ambient temperature is about 297.25 K. The bottom of the work units was coated with conductive silicone paste that contacts the heating platform to reduce the effect of thermal contact resistance. Lead wires at both sides of the thermoelectric legs act as positive and negative sides of the work units so that thermal electromotive force can be recorded. The resistance box was used as a load resistor to match the internal resistance of the work units for testing the output power. Four samples were prepared for each model and the test results were averaged. Repeated tests were performed on each sample to avoid errors.

## 5. Results and Discussion

Thermal regulation performance of Type-I, Type-II, and Type-III was evaluated. Simulation results of effects of encapsulated structure, the height of the thermoelectric single leg, heat dissipation area, and heat input temperature appear in Section 5.1, Section 5.2, Section 5.3 and Section 5.4. Experimental results of the work units of thermal electromotive force and output power are discussed in Section 5.5.

### 5.1. Effect of Encapsulated Structure

**Figure 6:**
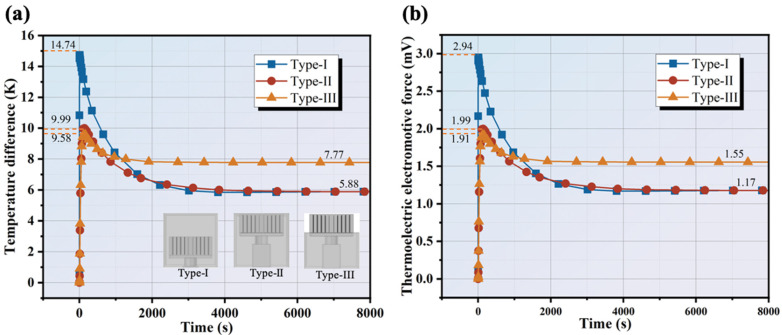
(**a**) Temperature difference; (**b**) Thermal electromotive force of Type-I, Type-II, and Type-III with different encapsulated structures.

It can be observed from Figure 6a that similar temperature difference trends exist in Type-I, Type-II, and Type-III. The maximum temperature differences are 14.74 K, 9.99 K, and 9.58 K for Type-I, Type-II, and Type-III, respectively, at 6.4 s, 139.2 s, and 157.9 s. This can be attributed to the high thermal conductivity of cement matrix which can abort and transfer the heat flow rapidly. In comparison, the maximum temperature difference of Type-II and Type-III is obviously smaller than that of Type-I because of the thermal resistance of the ceramic block. As Figure 6a indicates, the temperature difference in Type-I has an obvious downward trend in 3012 s, from 14.74 K to 5.95 K, reduced by 59.63% compared with the maximum temperature difference. The limited heat capability of the cement matrix can be well explained by the quick reduction of temperature difference. The amount of cement matrix in the vertical direction within Type-I is much more than that of Type-II so worse thermal regulation performance of Type-II was achieved, below 9.58 K. Nevertheless, it can be seen that Type-I and Type-II finally perform more poorly in terms of stable temperature difference (only 5.88 K) than Type-III (7.77 K) because of the finite heat exchange surface area. As the heat input and heat transfer gradually equalize, the temperature difference then stabilizes at 5.88 K under the condition of natural dissipation. Regarding the Seebeck effect, the corresponding thermal electromotive force’s overall trend is shown in Figure 6b. The stable thermal electromotive force is attained at 1.17 mV, 1.17 mV, and 1.55 mV, respectively, for Type-I, Type-II, and Type-III. The good power generation potential of Type-III can be seen.

In the literature [21], Cai also explored the dynamic change relationship of temperature difference between the two sides of a TEG in the STERB-CPCM model with radiant heat flux of 1000 W/m^2^ and convective heat transfer coefficient of 5 W/(m^2^·K) and found that the highest temperature difference was only 3.07 K, far lower than 7.77 K in this work. Meanwhile, the output power in [21] also shows a similar trend to the thermoelectric electromotive force presented in this section. Lin et al. [6] found that a TEG in a window frame can maintain a temperature difference of 6 K under the 25 K temperature difference between the indoors and outdoors. Meanwhile, Type-I and Type-II have a 5.88 K temperature difference under a 15 K ambient temperature difference in this work. This also shows that the composite structure of cement-based materials integrated with aluminum heatsinks has great potential for TEG heat dissipation.

The differentiation of thermal electromotive forces can also be explained by the position of the thermoelectric single leg, the thickness of the ceramic bulk, and the effect of the cement matrix. Because of the fast heat transfer of the cement matrix, Type-I has the best power generation ability in the earlier stage up to about 1000 s. However, the thermal electromotive force decreases quickly in a later stage with the increased temperature of the cement matrix. Type-II has a lower maximum temperature difference than Type-I due to the thermal resistance of ceramic block. Since the temperature is a gradient distributed inside the work units, the temperature differences of Type-I and Type-II converge at a steady state. Meanwhile, Type-III displays outstanding thermal regulation performance whose thermal electromotive force is improved by 32.14% compared with that of Type-I and Type-II. Hence, Type-III can be potentially used in the thermal regulation of TEG systems for the synergy effect of cement matrix and finned aluminum heatsinks.

**Figure 7:**
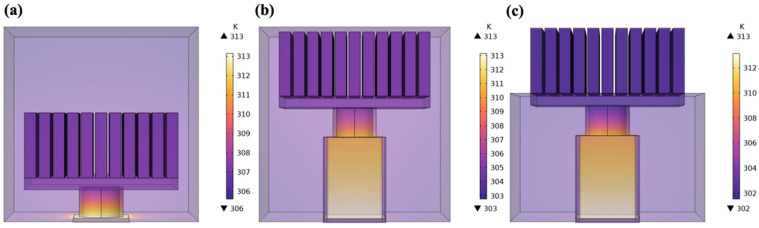
Temperature distribution of (**a**) Type-I; (**b**) Type-II; and (**c**) Type-III (Time = 8000 s).

Structure makes a difference in thermal regulation performance for work units. Figure 7a–c present the temperature distribution of different encapsulated structures at 8000 s. It can be observed that the lowest temperatures of Type-I, Type-II, and Type-III are about 306 K, 303 K, and 302 K. Type-II has a comparatively small amount of cement paste but lower overall temperature than Type-I due to the effect of the ceramic block which results in a lower cold side temperature of the thermoelectric single leg. For Type-III, its overall temperature is somewhat lower than that of Type-I and Type-II. Type-III can rely on cement-based material to transfer heat downward and rely on finned aluminum heatsinks to radiate heat to the surrounding environment to maintain a large and stable temperature difference.

As mentioned above, Type-I has a high instantaneous power generation capacity, which means that the problem of instantaneous thermal stress needs to be considered. The rational use of increasing the thermal resistance in the heat transfer process for Type-II and Type-III can stabilize the power generation process without affecting the steady-state electrical output capacity. For example, in the literature [25], Li et al. also pointed out that adding a heat pipe could further reduce the heat source temperature and regulate the TEG output performance. Due to the excellent heat dissipation capacity of Type-III, its minimum temperature is only 302 K, which is because the heatsink has a low thermal resistance, improving the heat dissipation performance of spreading heat to the environment. Similar composite structures can effectively achieve TEG thermal management and reduce the overall temperature of the system. Rezania et al. [13] also observed this phenomenon at 3600 s and under the heat load with on/off duration of 240 s. The temperature difference was improved 19.3% by implementation of the metal foam in the PCM heatsink.

### 5.2. Effect of Thermoelectric Leg Height

**Figure 8:**
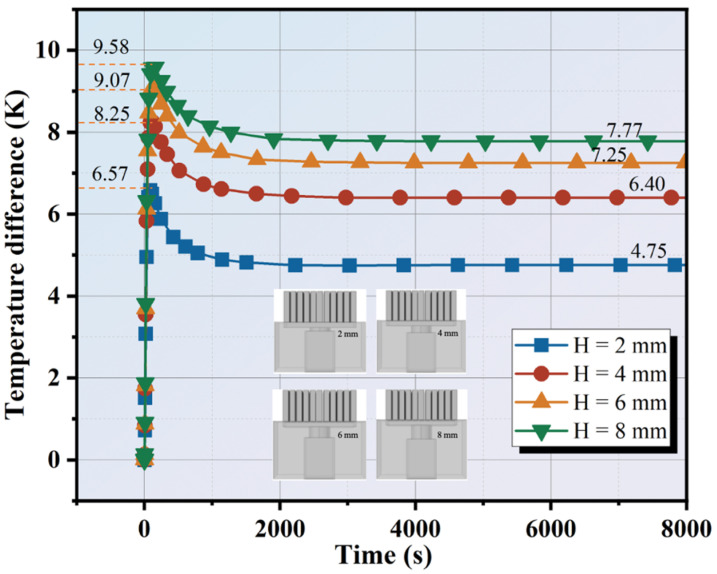
Temperature difference variation of Type-III with thermoelectric single leg height.

The height (H) of the thermoelectric single leg is of great importance for thermoelectric work units’ thermal regulation performance according to Fourier’s law. To analyze the effect of the height of the thermoelectric single leg on work units in practical applications, the heights are set as 2 mm, 4 mm, 6 mm, and 8 mm. The corresponding work units are established and simulated. The heat input temperature and ambient temperature are 313.15 K and 298.15 K, respectively. The finned aluminum heatsink is used. The temperature difference result of Type-III is shown in Figure 8, and the maximum temperature difference is about 9.58 K, 9.07 K, 8.25 K, and 6.57 K for thermoelectric single legs of 2 mm, 4 mm, 6 mm, and 8 mm. After about 8000 s, the temperature differences are maintained at 4.75 K, 6.40 K, 7.25 K, and 7.77 K. Compared with the height of 2 mm, the temperature differences are improved by 34.73%, 52.63%, and 63.57%, respectively, for 4 mm, 6 mm, and 8 mm. Obviously, the temperature difference is positively correlated with the height of the thermoelectric single leg, which means more height achieves a higher stable temperature difference. However, the temperature difference does not increase by a strict multiple. On the one hand, this is a consequence of the fact that part of the heat loss of the thermoelectric single leg during the heat transfer process cannot be used exclusively for the build-up of the temperature difference. On the other hand, this can be attributed to the heatsink having a limited rate of transferring heat to the cement matrix. In addition, the larger height of the thermoelectric single leg results in bigger thermal resistance which reduces heat transfer from the hot side to the cold side. At the later steady state, the cold side temperature of the thermoelectric single leg decreases with increasing height of the thermoelectric single leg. During the heat transfer process, the heat on the cold side accumulates the temperature increases, and gradually the temperature difference decreases. It implies that the temperature difference driving force is weakened and the heat transfer rate is reduced. Finally, the internal solid heat transfer and surface heat exchange reach an equilibrium state, and the temperature difference tends to balance.

Large thermoelectric leg height impedes the transfer of heat between the TEG and the heatsink. Nevertheless, increasing the height to maintain the temperature difference is of limited use. Although increasing the height can increase the thermal resistance and widen the temperature difference, it also means a significant increase in the thermoelectric material cost. Cai et al. further established a model considering system performance and operating cost and demonstrated that the height of the thermoelectric leg has a direct relationship with heat conduction and cost [26]. The appropriate material parameters should be determined according to the actual application scenario.

### 5.3. Effect of Heat Dissipation Area

**Figure 9:**
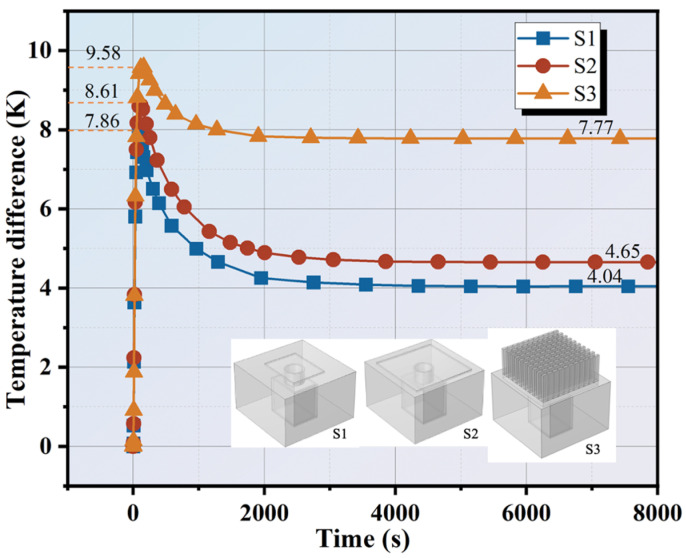
Temperature difference variation of Type-III with heat dissipation area.

The cement matrix does not possess the flow characteristics of liquids. Consequently, an additional heatsink area of the work unit is an essential way to dissipate heat quickly. To clarify the impact of the heat dissipation area on the temperature difference, we use three different heatsinks for discussion. As illustrated in Figure 9, the numerical simulation reveals the effect of the heatsink area of S1 (22 mm × 28 × 1 mm), S2 (40 mm × 40 × 1 mm), and S3 (finned aluminum heatsink) for Type-III. The physical heatsinks can be referenced in Figure 2b. The height of the thermoelectric single leg is 8 mm. The heat input temperature is 313.15 K and the ambient temperature is 298.15 K. For S1, S2, and S3 units, the temperature increases to the maximum value of about 9.58 K, 8.61 K, and 7.86 K in the initial 2000 s. The reason for this result is that the thermal capability of the cement matrix is low. Therefore, in this stage, the heat transfer is mainly dominated by heat absorption of cement-based material. Finally, the temperature differences become stable at 4.04 K, 4.65 K, and 7.77 K, respectively. Due to the large exchange area with air, the S3 unit has the maximum temperature difference of 7.77 K which is improved by 92.32% and 67.09% compared to that of S1 and S2.

A larger heat dissipation area can quickly transfer the heat from the cold side to the cement matrix and ambient environment. This accelerates the transfer of heat from the cold side, slows down the effect of heat stasis, and maintains a stable temperature difference between the two sides for a long time. The larger the heat dissipation area, the lower the temperature at the cold side of the corresponding work unit, which indicates that the effectiveness of heat dissipation is more distinct. Increasing dissipation area corresponds to an improvement in the average thermal conductivity of the overall composite structure. Meanwhile, the cement matrix has a high thermal conductivity which enhances the heat transfer rate in the initial stage. Importantly, increasing the area of heat exchange with the air is more effective in maintaining the temperature difference, as the S3 unit result indicates. In addition, owing to the huge contact area between the heatsink and the cement matrix, as well as air, the thermal management performance of the S3 unit is much superior to that of S1 and S2. A large dissipation area is conducive to heat transferring from the cement matrix inside to the surface, improving the thermal management performance. On the one hand, it increases the contact area with cement-based materials. On the other hand, it increases the heat exchange area with the surrounding environment and transfers the heat as soon as possible.

Li [25] increased the contact area between heat-dissipating metal and PCM by changing the porosity of porous foam, 0.82 and 0.96, and found that the TEG voltage output of 0.96 porosity was significantly better than that of 0.82, and the TEG thermal management temperature of 0.96 porosity was slightly lower than that of 0.82. This trend is consistent with the results shown in this section by changing the heat dissipation area. Similarly, adding the metal foam can reduce the thermal resistance of the system regarding heat dissipation, thereby increasing the heat flow of the heat storage material [27].

### 5.4. Effect of Heat Input Temperature

**Figure 10:**
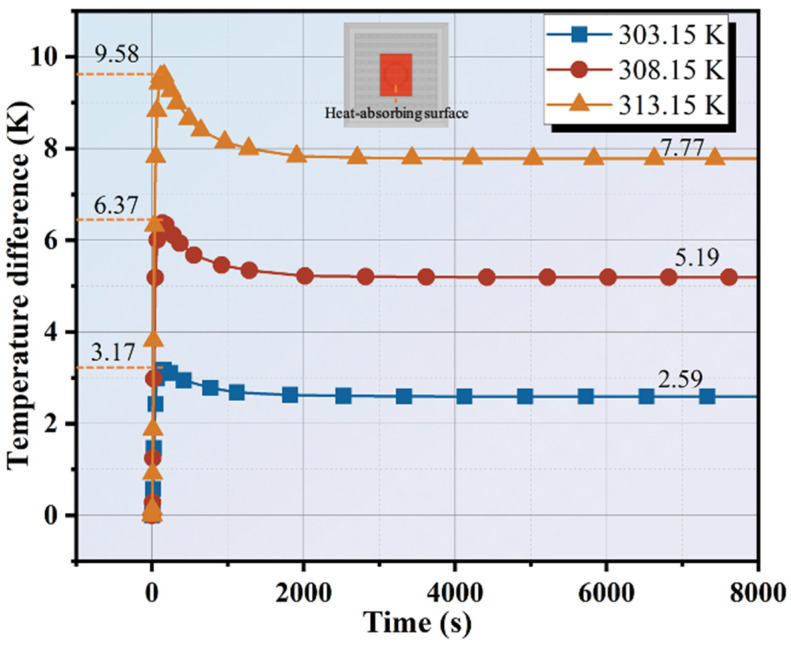
Temperature difference variation of Type-III with heat input temperature.

The diameter and height of the thermoelectric single leg in this section are 10 mm and 8 mm, respectively. The ambient temperature is 298.15 K and the finned aluminum heatsink is used. The numerical simulation investigates the effect of heat input temperatures of 303.15 K, 308.15 K, and 333.15 K, respectively, on the Type-III thermal regulation performance. As illustrated in Figure 10, the temperature difference between the cold side and hot side variation with heat input temperature is presented. Based on the Seebeck effect, the thermal electromotive force is positively proportional to the temperature difference. Hence, the output performance is enhanced by the heat input temperature. In the case of Type-III, the maximum temperature differences are 3.17 K, 6.37 K, and 9.58 K, respectively, for 303.15 K, 308.15 K, and 313.15 K. Subsequently, the reduced temperature difference for the heated cement matrix is equivalent to an additional thermal resistance that hinders thermal transfer. Finally, the stable temperature differences are 2.59 K, 5.19 K, and 7.77 K, respectively. Mathematically, the temperature difference of Type-III for input temperature of 313.15 K represents an improvement of 49.71% and 200% compared with that of 308.15 K and 303.15 K. It is evident that the greater the difference between the heat input temperature and ambient temperature is, the greater the heat flux and temperature difference are. Also, there are two gentle stages in the curves, which correspond to the cement matrix heat absorption and finned aluminum heatsink heat exchange. Cai also [21] simulated the temperature difference of a TEG (with two PCM blocks on its two sides) with solar radiation of 600 W/m^2^, 800 W/m^2^, and 1000 W/m^2^, but the maximum temperature difference is 1.78 K, 2.33 K, and 2.87 K, respectively, which is far lower than that of this work. It displayed a similar trend. However, in some long-term high-temperature conditions, it is necessary to consider the influence of thermal stress [28].

It has been observed from the simulation results mentioned above that the encapsulated structure has a significant impact. The models used in the study predict that Type-III can achieve an optimized temperature difference of 7.77 K. The significant increase in temperature difference is due to the extensive heat exchange area of the finned aluminum heatsink. Further evaluation of the height of the thermoelectric single leg and heat dissipation area revealed that an increase in these parameters, as well as the heat input temperature, led to an improvement in temperature difference. This is because these parameters are directly related to thermal resistance and the heat transfer process. Reasonable regulation of these parameters has a positive significance for improving indoor temperature and comfort. Especially, the Type-III model demonstrates the essential potential for thermoelectric work units encapsulated with cement paste, which can be hopefully used for waste heat recycling in building surface energy harvesting.

### 5.5. Experimental Results

**Figure 11:**
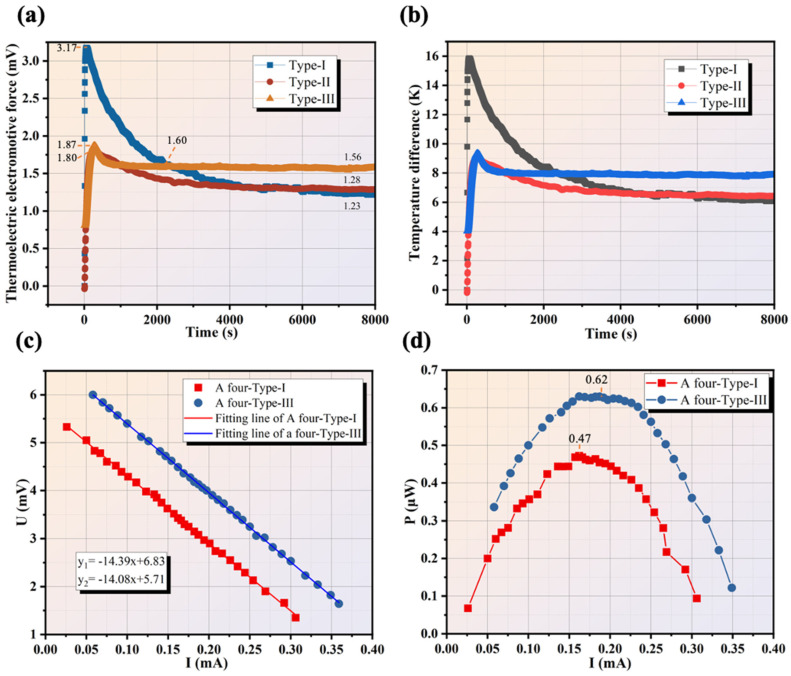
Output performance of work units at input temperature of 313.15 K: (**a**) Thermal electromotive force; (**b**) Temperature difference; (**c**) Volt–ampere characteristics of a four-Type-I and a four-Type-III; (**d**) Output power of a four-Type-I and a four-Type-III versus current.

To see the actual generation capacity of the thermoelectric work units, experiments for testing thermal electromotive force and output power were conducted in 8000 s. We used 42.5 ordinary Portland cement only, with a W/C of 0.3, to fabricate encapsulating cement paste as the cement-based material and encapsulate the TEG structures. Figure 11a shows the response temperature variation for the three work units with different TEG structures. In the earlier stage, heat transfer is primarily in the form of heat conduction. The numerical results qualitatively correspond with experimental findings, but the real-time thermal electromotive force has some relative errors. In the case of Type-I, it takes 142 s to reach the instantaneous maximum value (3.17 mV) and this quickly diminishes to about 1.60 mV at 2334 s, which can be attributed to the rapid heat transfer of the cement matrix. The hardened cement paste has a dense structure and high thermal conductivity (>1 W/(m·K)) [29], where cement matrix absorbs in the short term due to its finite thermal capability. Also, the large heat exchange area of the finned aluminum heatsink enhances the heat transfer process. On the other hand, part of this can be illustrated by the endothermic capability of inside free water of the cement matrix. Afterward, a small loss of thermal electromotive force was observed from 1.60 mV to 1.23 mV, which declined by 23.12%. In the case of Type-II, it takes about 260 s to reach the maximum value (1.80 mV). It was then decreased to 1.39 mV in 1800 s. Finally, it stabilizes at about 1.28 mV. For Type-III, it takes about 280 s to reach the maximum value (1.87 mV). It then decreases to 1.6 mV in 800 s.

In the steady state, the thermal electromotive force stabilizes at 1.56 mV. The upper part is a finned aluminum heatsink with a large surface to exchange heat with air. The heat conduction of the cement matrix plays a dominant role in the first 800 s. Then, the solid heat conduction and radiation of the cement matrix surface and the radiation of the heatsink function together. This is also in good agreement with the simulation results. A more stable thermal electromotive force can be maintained for a longer time for Type-III compared with Type-I and Type-II. The real-time temperature difference can be calculated by Equation (8) [30], as shown in Figure 11b.

(8) $$E_{oc}=N\alpha (T_{h}-T_{c})$$

where *N* is the number of thermoelectric single legs, *T_h_* and *T_c_* represent, respectively, the hot side and cold side temperature of thermoelectric single leg, and the difference between the two is ∆T. In general, Type-III has the ability to maintain a high temperature difference, and its stable temperature difference is 26.82% higher than that of Type-I and Type-II.

The thermal electromotive force ($E_{oc}$) of a four-Type-I assemblage and a four-Type-III assemblage are about 5.71 mV and 6.83 mV. As previously stated, the steady-state thermal electromotive forces of Type-I and Type-II are identical. Therefore, the main discussion is on the output performance of Type-I and Type-III. Figure 11c shows volt–ampere characteristics of a four-Type-I and a four-Type-III in approximately 8000 s.

According to Olm’s law, the output performance can be derived by Equations (9)–(11) [30]:

(9) $$U_{L}=E_{oc}-IR_{in}=IR_{L}$$

(10) $$I=\frac{E_{oc}}{R_{in}+R_{L}}=\frac{N\alpha \Delta T}{R_{in}+R_{L}}$$

(11) $$P=I^{2}R_{L}={\left(\frac{N\alpha \Delta T}{R_{in}+R_{L}}\right)}^{2}\times R_{L}$$

where $U_{L}$ is the voltage difference of load resistance on two sides, *I* is current, $R_{in}$ is the internal resistance of work units, and $R_{L}$ is the load resistance. The matched resistance value is of great importance and gives indications of the work unit output performance. The electric equipment is connected to the work units and the maximum energy conversion can be achieved if the matched resistance condition is met. As shown in Figure 11c, it can be observed that the load resistance is about 14 Ω when the maximum output power is measured. The inner resistance of work units is not that much. Because the external resistance includes circuit contact resistance, the resistance of the resistor box and ammeter is ~13 Ω, which increases the external resistance and diminishes the output power. This is caused by objective testing errors and data acquisition in the experimental system. As shown in Figure 11d, the output power and current of a four-Type-I assemblage produces the maximum value of 0.47 μW, and a four-Type-III assemblage produces the maximum value of 0.62 μW at steady state. Although the four thermoelectric units in series only have a microwatt level of electrical power output, in practical use, commercial TEG modules can be used to achieve P-N series at the level of hundreds of legs to achieve large-scale energy collection.

The cement-based materials used for TEG heat dissipation are feasible. In heat disspation, Lee [7]proposed the used of the road concrete structures (surface and interior) for thermoelectric energy collection, achieving a long-term stable voltage output above 0.06 V. Due to the rapid heat dissipation ability of cement-based materials, they have great potential for heat dissipation in building TEG systems. Moreover, cement-based composite materials with added expanded graphite and metal oxide have higher thermal conductivity and can also reduce the surface temperature (1–3 °C) and even effectively alleviate the urban heat island effect [17]. For TEG systems with a composite structure of cement-based material integrated with metal heatsinks, phase change cement-based materials with high thermal conductivity and heat capacity should be developed with the aim of enhancing the heat dissipation capacity of the TEG and improving comfort inside the building.

## 6. Conclusions

In this experiment, the effect of the composite structure of cement-based material integrated with heatsinks on the heat dissipation capacity of thermoelectric work units is studied. The relationship between the encapsulated structure, the height of the thermoelectric leg, the heat dissipation area, and the heat input temperature and the maintained temperature difference is shown in the numerical simulation. The effect of the encapsulated structure on heat dissipation of thermoelectric work units is verified experimentally.

The results show that cement-based materials have significant potential for heat dissipation in thermoelectric units. For Type-I and Type-II, the lowest temperature difference is maintained at 5.88 K. Meanwhile, the composite structure of the cement-based material and the heatsink is also a key factor in the heat transfer mechanism and maintaining the temperature difference of the TEG system. In this study, the cement-based material provides heat storage and heat conduction, while the heatsink also improves the heat transfer of the thermoelectric work units. Combined with the advantages of cement-based materials’ rapid heat conduction and the high heat exchange capacity of the aluminum heatsink, the temperature difference can even be maintained at 7.77 K. In general, in the case of transient heat load, Type-I can achieve fast work and provide higher power output, but it also needs to solve the problem of instantaneous thermal shock. Type-II is deeply embedded in the building structure, and under the same thermal conditions, it can achieve the same level of power generation as Type-I at a steady state. Remarkably, Type-III has the ability of Type-II to stabilize and maintain a high temperature difference, and its stable temperature difference is 32.14% higher than that of Type-I and Type-II and increased by 26.82% in the actual experiment. Furthermore, the simulation results show that the height of the thermoelectric leg, the heat dissipation area, and the heat input temperature are positively correlated with the temperature difference. This work provides a reference for structural composites of cement-based materials integrated with heatsinks to be applied to the heat dissipation of TEG systems for building energy harvesting.

## Figures and Tables

**Table 1:** Dimensions of finned aluminum heatsink.

Parameter	Value (mm)
Dimension	40 × 40 × 20
Number of fins	110
Fin width	3
Fin thickness	1.5
Fin height	17
Channel width	2.75

**Table 2:** Thermoelectric Properties of P-type Bi_2_Te_3_.

Performance Specification	P-Type Bi_2_Te_3_	Temperature (K)
Electrical conductivity (10^2^ × S/m)	2000~2600	300~600
Seebeck coefficient (μV/K)	≥140	300~600
Thermal conductivity (W/(m·K))	≥1.5	300~600

**Table 3:** Physical Properties of Materials.

Category	Ceramic	Epoxy Resin	Foam Insulation	Al Finned Heatsink	Cement Matrix
Density (kg/m^3^)	3900	1673	24	2700	2300
Thermal conductivity (W/m/K)	27	0.44	0.023	238	1.8
Specific heat capacity (kJ/(kg·K))	0.9	0.55	0.002	0.9	0.88

## Data Availability

Data are contained within the article.
